# COVID-19 in Elderly Adults: Clinical Features, Molecular Mechanisms, and Proposed Strategies

**DOI:** 10.14336/AD.2020.0903

**Published:** 2020-12-01

**Authors:** Ya Yang, Yalei Zhao, Fen Zhang, Lingjian Zhang, Lanjuan Li

**Affiliations:** State Key Laboratory for Diagnosis and Treatment of Infectious Diseases, National Clinical Research Center for Infectious Diseases, Collaborative Innovation Center for Diagnosis and Treatment of Infectious Diseases, The First Affiliated Hospital, College of Medicine, Zhejiang University, Hangzhou, China.

**Keywords:** COVID-19, elderly, clinical feature, molecular mechanism, strategy

## Abstract

Coronavirus disease 2019 (COVID-19) is causing problems worldwide. Most people are susceptible to severe acute respiratory syndrome coronavirus 2 (SARS-CoV-2), but elderly populations are more susceptible. Elevated susceptibility and death rates in elderly COVID-19 patients, especially those with age-related complications, are challenges for pandemic prevention and control. In this paper, we review the clinical features of elderly patients with COVID-19 and explore the related molecular mechanisms that are essential for the exploration of preventive and therapeutic strategies in the current pandemic. Furthermore, we analyze the feasibility of currently recommended potential novel methods against COVID-19 among elderly populations.

Coronavirus disease 2019 (COVID-19) is an emerging respiratory infectious disease caused by severe acute respiratory syndrome coronavirus 2 (SARS-CoV-2), which was first identified in mid-December 2019 in Wuhan, China. Currently, it has spread globally and been declared a pandemic by the World Health Organization (WHO). Up to August 18,2020, more than 767,000 deaths and 21,500,000 confirmed positive cases have been reported around the world. Accumulating evidence shows that age and underlying conditions of virus contacts are crucial factors influencing personal fate toward different clinical severities of COVID-19, ranging from asymptomatic, mild, and moderate to death [1, 2]. COVID-19 shows a considerably elevated mortality rate in patients with advanced age and pre-existing comorbidities [3]. Under the situation of global aging, the COVID-19 pandemic creates challenges for not only widespread public health but also biomedical and clinical aging research [4].

## Clinical features of COVID-19 in elderly patients

New coronavirus-exposed populations are generally susceptible, but elderly people, especially those with underlying conditions normally considered to be aging-associated diseases (diabetes, hypertension, and cardiovascular and cerebrovascular diseases), exhibit increased susceptibility [5]. Moreover, the spread of SARS-CoV-2 as well as severe acute respiratory syndrome coronavirus (SARS-CoV) in 2003 made it clear that these aging patients are more likely to progress to severe disease and die more easily from these infections than younger people [6-8]. After analyzing the data for 1099 COVID-19 patients from 552 hospitals in China, Nanshan Zhong *et al*. found that patients with severe disease were older than those with nonsevere disease and that coexisting illness was more common among patients in the severe group[9]. In the United States, 80% of deaths have occurred in patients over the age of 65 years, and patients aged 85 years and over have a high proportion of severe outcomes, mirroring the experience in China [10]. According to a meta-analysis, serious aging-related chronic diseases(associated with a pathological role of cellular senescence), including hypertension, diabetes, chronic obstructive pulmonary disease (COPD), cardiovascular disease, and cerebrovascular disease, are independent risk factors associated with COVID-19 patients [11].

Similar to those in younger patients, fever, cough and sputum are the most common early symptoms in elderly patients. Elderly patients are more likely to have severe COVID-19 conditions, show lack of improvement, and die [12]. Analyzing a total of 56 COVID-19 patients, Liu *et al*. found that the pneumonia severity index (PSI) score of elderly patients was higher than that of young and middle-aged patients. The proportion of patients with PSI grades IV and V was significantly higher in elderly patients [13]. The incidence of the common fatal presentations of COVID-19 (including ARDS, shock, arrhythmia and acute cardiac injury) in elderly patients is higher than in young patients [12, 14, 15]. Wang *et al*. included patients with laboratory-confirmed COVID-19 and ascurtained the viral load by reverse transcriptase quantitative PCR (RT-qPCR). The results showed that older age was correlated with higher SARS-CoV-2 load [16]. Studies of SARS-CoV have shown that a high initial viral load is associated with death [17]. Accumulating evidence indicates that the SARS-CoV-2 viral load correlates with the risk of COVID-19 progression and poor prognosis [18, 19]. Thus, it is conceivable that the poor prognosis of elderly COVID-19 patients may be related to a higher viral load. However, the underlying mechanism requires further exploration.

## Mechanism of COVID-19 in elderly patients

Senescent cells accumulate with age in vertebrates and promote aging mainly through the senescence-associated secretory phenotype (SASP) [20]. Data have shown that RNA viruses, such as influenza virus, display enhanced replication efficiency in senescent cells, which suggests that the accumulation of senescent cells in aging and age-related diseases may play a role in this phenomenon. However, the response to SARS-COV-2 that occurs in senescent cells is still poorly understood [8].

Angiotensin-converting enzyme 2 (ACE2) has been identified as areceptor for SARS-CoV-2, which directly interacts with COVID-19 spike(S) glycoproteins [21, 22]. During infection, the S protein is cleaved into S1 andS2 by the host transmembrane protease/serine subfamily member 2 (TMPRSS2)and protease furin [23].S1 directly binds to ACE2 and S2 plays a role in membrane fusion [24]. Zhou et al. analysed the expression of ACE2, together withTMPRSS2 and Furin by the method of single-cell RNA profiling combined with the protein information in different tissues. According to the rank list of candidate cells potentially vulnerable to SARS-CoV-2,the top targets were lung alveolar type 2 (AT2) cells and macrophages, followed by cardiomyocytes and adrenal gland stromal cells [25]. These findings were consistent with prominent lung symptoms, frequent heart damage and rare bowel symptoms. Cluster of differentiation 26 (CD26) is also recommended as a potential receptor for SARS-COV-2 [22, 26]. Intriguingly, both ACE2 and CD26 show associations with senescence and immunoregulation.

ACE2, which is widely distributed in the heart, kidneys, lungs, liver, intestine, brain and testes, is a known crucial component of the renin-angiotensin system (RAS)[27]. According to Khemais-Benkhiat *et al*., RAS is upregulated in endothelial cells with premature and replicative senescence [28]. It has been suggested that differential levels of ACE2 in human organs, especially the cardiac and pulmonary tissues of younger versus older adults, may be responsible for the disease virulence spectrum observed in COVID-19 patients [29]. Ziegler *et al*. discovered that ACE2 was a human interferon-stimulated gene in airway epithelial cells. SARS-CoV-2 could enhance infection through species-specific interferon-driven upregulation of ACE2 [30]. Smith *et al*. also identified ACE2 as an interferon-stimulated gene in lung cells. They found that chronic smoke exposure and inflammatory signaling could increase ACE2 expression levels in the respiratory tract. Their findings suggested that SARS-CoV-2 infections could create positive feedback loops that increase ACE2 expression and promote viral dissemination [31].

Interestingly, it has been previously established that ACE2 shows a protective effect in the lungs and that ACE2 expression in the lungs decreases during aging. The lower ACE2 level in the lungs may contribute to the poor prognosis of SARS in the aged group [32]. According to previous research, following SARS-COV entry, ACE2 expression is downregulated dramatically, which leads to a compensatory overproduction of angiotensin 2 (AngII), therefore increasing lung vascular permeability and aggravating lung failure [33]. A similar phenomenon is also observed in SARS-CoV-2-infected lung tissue [34]. According to Datta *et al*., SARS-CoV-2infects ACE2-expressing cells in the lung, inducing shedding of ACE2 from the cells and, thus, effectively reducing ACE2 expression levels [35]. These findings support the proposed role of ACE2 downregulation in both the pathogenesis and progression into ARDS in SARS and COVID-19 patients.

The contradictions between these research results indicate that SARS-CoV-2 regulation of ACE2 may be complex and dynamic. Whether the regulatory mechanism of SARS-CoV-2 toward ACE2 differs due to the patients’ age, underlying diseases, and stages of infection requires further exploration.

ACE2 is enriched in the heart and plays an essential role in the regulation of heart function [36]. Burrell *et al*. indicated that ACE2 expression is increased in myocardial infarction in humans and rats [37]. Chen *et al*. performed state-of-the-art single-cell atlas analysis of the adult human heart and revealed pericytes with high expression levels of ACE2 as target cells of SARS-CoV-2. Patients with coronary heart disease (CHD) showed increased ACE2expression at the mRNA and protein levels [38]. Patients with CHD and infected with SARS-CoV-2 are at an elevated risk of progressing into severe disease [11, 29]. Conversely, acute cardiac injury is reported as one of the most common complications in COVID-19 patients, which significantly exacerbates disease severity [39]. This evidence suggests that enrichment of ACE2 in the myocardium makes the heart one of the main target organs for SARS-CoV-2 infection. The expression level of ACE2 in cardiomyocytes is closely related to the poor prognosis of COVID-19 patients with heart diseases and the severity of COVID-19-induced heart injury.

Cytokine storms, namely, hypercytokinemia, a frequent feature of severe SARS, MERS, H5N1 influenza and H7N9 influenza, are associated with disease severity and are a predictor of prognosis [40-42]. Accumulating data suggest that depletion of lymphocytes, activation of cytotoxic T-lymphocytes, neutrophils and neutrophil-mediated cytokine storms may play key roles in COVID-19 pathogenesis [43]. COVID-19 results in increased levels of plasma cytokines, including interleukin-6 (IL-6), interleukin-1β (IL-1β), interleukin-7 (IL-7), interleukin-8 (IL-8), interleukin-9 (IL-9), interleukin-10 (IL-10), granulocyte colony stimulating factor (G-CSF), granulocyte-macrophage colony-stimulating factor (GM-CSF), interferon-γ (IFN-γ), tumor necrosis factor (TNF-α) and vascular endothelial growth factor A (VEGFA). ICU patients with COVID-19 produced higher levels of IL-6 than those not requiring ICU care [44]. According to a meta-analysis, mean IL-6 serum levels are2.9 times higher in patients with severe disease than in those with nonsevere disease [45]. HighIL-6 levels are closely correlated with the serum SARS-CoV-2 viral load, which further affects vital signs and the occurrence of ARDS inCOVID-19 patients [46]. Moreover, as a mediator of SASP, IL-6 is the main functional marker of cell aging [5, 47], and the increase in IL-6 content may correspond to the age-dependent increase in mortality of COVID-19 patients [48].

ACE2 participates in the RAS and plays an important role in regulating IL-6-induced inflammatory injury through the NF-κB and STAT3 pathways [49]. Kuba*et al*. indicated that SARS-CoV infection could cause a reduction of ACE2 levels on cells, followed by increased serum AngII levels [50]. AngII is one of the key activators of NF-κB and STAT3, which stimulates the expression of IL-6[51]. In turn, we speculate that severe lung inflammation induced by SARS-CoV-2 infection may induce the dysregulation of the RAS followed by the development of ARDS.

CD26, also known as dipeptidyl peptidase 4 (DPP4), is a serine exopeptidase that is expressed ubiquitously in human tissues, including lung, kidney, liver, gut, and immune cell s(including T-cells, activated B-cells, activated natural killer cells and myeloid cells) [52, 53]. CD26 plays multiple roles in nutrition, metabolism, and the immune and endocrine systems. Using mass spectrometry analysis, Kim *et al*. identified CD26 as a surface protein that is expressed at significantly higher levels in senescent cells. CD26 activity is higher in older than in younger individuals [54]. Overly upregulated CD26 expression and activity are associated with diabetes, obesity and metabolic syndrome, which are reported to influence COVID-19 severity [22, 55].

Moreover, CD26 has been identified as the functional receptor of MERS-CoV [56, 57]. Research has indicated that MERS-CoV is associated with higher mortality among elderly populations with chronic debilitating diseases including diabetes, malignancy, pulmonary and renal diseases. MERS also appears with greater severity and higher mortality rates in elderly people, especially those with debilitated immune systemsorpre-existing comorbidities [58-60]. Therefore, we suggest that similar to the case for ACE2, DPP4 upregulation may be adeterminant of COVID-19 progression and prognosis.

Large numbers of studies have demonstrated the integral role of CD26 in T lymphocyte activation during immunesenescence and costimulation [61]. CD26 acts as a costimulatory agent that mediates T cell activation by binding to the ligand adenosine deaminase [62]. Additionally, CD26 enhances lymphocyte proliferation independent of ADA [63].

**Figure1.**
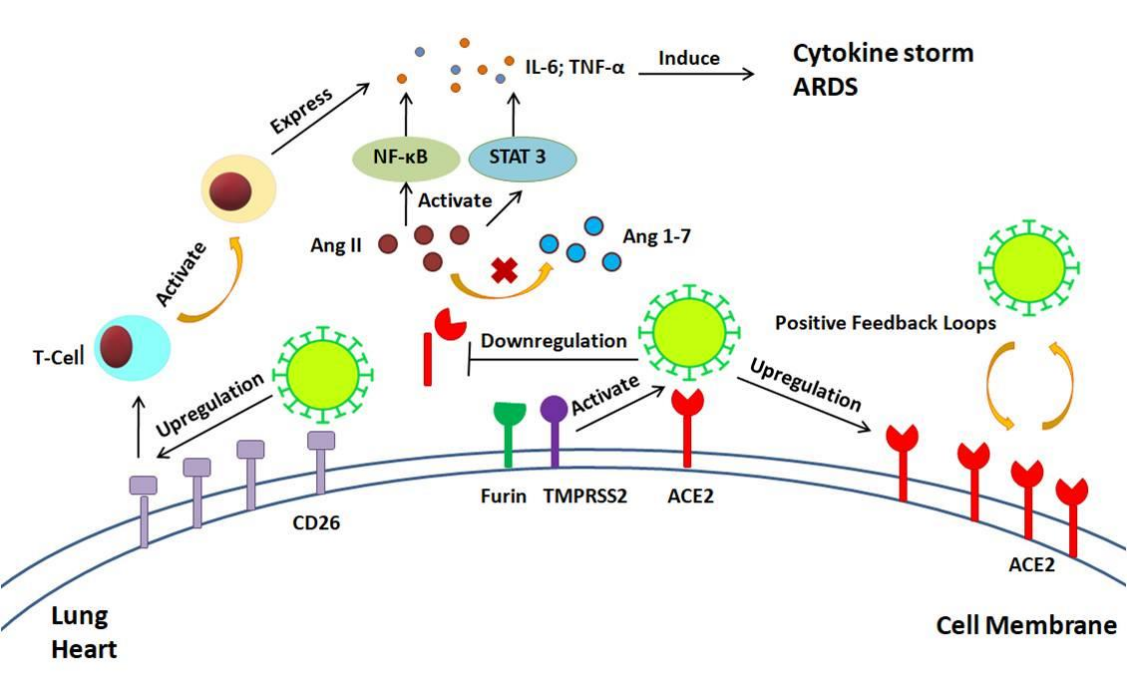
Interaction of SARS-CoV-2 with ACE2 and CD26. To enter the host cells, SARS-CoV-2 binds to membrane-bound ACE2 with the assistance of Furin and TMPRSS2. SARS-CoV-2 infections could create positive feedback loops that increase ACE2 expression and promote viral dissemination. On the other hand, SARS-CoV-2 infections may induce ACE2 shedding. ACE2 downregulation could lead to accumulation of Ang II, therefore inducing cytokine storm and ARDS. Activation of CD26 on T lymphocytes may partially contribute to the high expression of IL-6 in COVID-19 patients.

CD26 can exist in a soluble form that is considered to be released from the membrane into the plasma, which still retains its enzymatic activity[64]. Ikeda *et al*. found that soluble CD26 enhances the binding of transcription factors to the TNF-α and IL-6 promoters, thus increasing TNF-α and IL-6 mRNA and protein expression in THP-1 cells [65]. CD26 inhibitors decrease the concentration of proinflammatory factors, such as TNF-α and IL-6 [66]. Thus, some researchers have suggested that the activation of CD26 on T lymphocytes may partially contribute to the high expression of IL-6 in COVID-19 patients. However, the specific role of CD26 in COVID-19 still requires further exploration (Figure 1).

## Proposed clinical applications in elderly covid-19 patients

Current evidence suggests that elderly patients with past comorbidities or dyspnea should be closely monitored for signs of disease progression, decompensation, and exacerbation of illness, especially 1-2 weeks after the onset of symptoms[10, 67]. Presently, there are no officially approved medicines available against SARS-CoV-2. Although the management of COVID-19 patients is primarily supportive, specific therapies are still under investigation. These therapies include current and potential antiviral drugs, anti-senescence drugs, immunosuppressive agents, steroids, mesenchymal stem cell (MSC) transplantation, and an artificial liver system (ALS), among others (Table 1). In the following paragraphs, we aim to highlight the proposed therapeutic strategies [68].

### Antiviral drugs

Timely antiviral therapy is highly recommended early the course of COVID-19 patients, especially elderly patients and patients with underlying conditions. Wu *et al*. retrospectively investigated the clinical data of 280 COVID-19 cases and recommended that timely antiviral treatment should be initiated to slow disease progression and improve prognosis in elderly patients [2]. Previously, oseltamivir, which could reduce the mortality of influenza patients, and ganciclovir, which is primarily used to treat cytomegalovirus [69], were widely used for SARS-CoV-2 patients. However, the efficiencies of neuraminidase inhibitors are currently being questioned, which makes them beyond recommendation [70, 71].

Remdesivir (GS-5734) is a monophosphoramidate prodrug of an adenosine analog that can affect viral RNA polymerase and reduce the production of viral RNA [72]. Remdesivir has shown a significant therapeutic effect in MERS-CoV mouse models [73]. It is effective in the EC50 range of 0.069 μM for SARS-CoV and 0.074 μM for MERS-CoV in tissue cultures as well as 0.77 μM in Vero E6 cells [72, 74]. Theoretically, remdesivir is currently the most promising drug for treating COVID-19. According to the result of a prospective, open-label study of remdesivir, Antinori *et al*. indicated that this drug could benefit hospitalized patients with SARS-CoV-2 pneumonia, with fewer adverse events observed [75]. The clinical trial conducted by Wang *et al*. in 237 patients at ten hospitals in Hubei Province showed that, although not statistically significant, the patients receiving remdesivir had a faster time to clinical improvement than those receiving placebo [76]. In the trial conducted by Beigel and colleagues, 1063 COVID-19 patients were randomly assigned to remdesivir or placebo. The results showed that remdesivir could obviously shorten the recovery time in hospitalized COVID-19 patients [77]. The authors also emphasized that earlier remdesivir treatment was probably more beneficial than later treatment [78]. Further explorations of the safety and efficiency of remdesivir for COVID-19 are still ongoing.

**Table 1:** Potential strategies for the treatment of COVID-19.

Treatment	Agent	Related Target/Pathways	Potential efficacy in COVID-19
Antiviral drugs	Remdesivir LPV/RTV Favipiravir Arbidol	Reduces the production of viral RNA Inhibits antiretroviral protease Targets RNA-dependent RNA polymerase Perturbs the virus membrane structure	Shortens the recovery time in COVID-19 patients Shortens the viral shedding duration in patients Induces a shorter viral clearance time and greater improvement rate in chest imaging Shorter duration of positive RNA test compared to those treated with LPV/RTV
Antisenescence drugs	Azithromycin Chloroquine; hydroxychloroquine Rapamycin	Targets and removes senescent cells; inhibits IL-6 and IL-1β expression; extends the lifespan of myofibroblasts Prevents the induction and accumulation of β-Gal; inhibits the replication of SARS-CoV *in vitro* Downregulates the IL-6 pathway; reduces the number of senescent T-cells through the mTOR-NLRP3-IL-1β axis	Reduces airway inflammation; antifibrosis Reduces the viral load in COVID-19 patients Prevents and treats the severity of COVID-19 patients
ACE2-related therapy	ACE2 activator ACE2 inhibitor Human recombinant soluble ACE2	Avoids binding of S protein of SARS-CoV-2 to ACE2 Inhibits ACE2 expression Directly binds to SARS-CoV-2 in the circulation	Requires scientific and clinical evidence Still under debate Blocks SARS-CoV-2 infection; prevents lung injury
CD26 inhibitor	Linagliptin	Attenuates DM-induced activation of NLRP3 inflammatory bodies	Decreases the concentration of cytokines, especially TNF-α and IL-6
Immunosuppressive Therapy	Tocilizumab; sarilumab; siltuximab cyclosporine-cyclophilin A complex Corticosteroids	Directly targets IL-6 receptors Halts the expression of TNF-α and IL-2; blocks the replication of coronaviruses Inhibits innate and adaptive immune responses as well as immune cells	Improves clinical outcomes in severe cases Anti-inflammatory and antiviral properties in COVID-19 Improves clinical outcomes in COVID-19 patients with ARDS
MSC transplantation	/	Advantages in anti-inflammation, antifibrosis and injury repair	Improves pulmonary function and symptoms of patients
Artificial liver system	/	Attenuates the cytokine storm	Reduces the mortality of severe patients exhibiting rapid disease progression

Abbreviations: COVID-19, coronavirus disease 2019; LPV/RTV, Lopinavir/ritonavir; IL-6, Interleukin-6; IL-1β, Interleukin-1β; β-Gal, beta-galactosidase; SARS-CoV, severe acute respiratory syndrome coronavirus; mTOR, mammalian target of rapamycin; NLRP3, nod-like receptor family pyrin domain-containing 3; ACE2, Angiotensin-converting enzyme 2; SARS-CoV-2, severe acute respiratory syndrome coronavirus 2; CD26, cluster of differentiation 26; DM, diabetes mellitus; TNF-α, tumor necrosis factor-α; IL-2, Interleukin-2; MSC, mesenchymal stem cell.

Lopinavir/ritonavir (LPV/RTV),which acts as antiretroviral protease inhibitor, is used as an anti-HIV drug [79]. In India, the Central Drugs Standard Control Organization approved the restricted public health use of the LPV/RTV combination in symptomatic COVID-19 patients [80]. However, the efficacy of LPV/RTV for COVID-19 is still controversial. A clinical trial in Hubei showed that early LPV/RTV treatment could shorten the viral shedding duration in COVID-19 patients, especially in those of an older age [81]. Cheng *et al*. obtained the exact opposite result among patients with mild pneumonia in Taiwan [82]. According to a randomized, controlled, open-label trial in 199 patients with severe COVID-19, mortality at 28 days and viral clearance time were similar in the LPV/RTV group and the standard-care group. The results suggested that no benefit was observed with LPV/RTV treatment [74]. Therefore, the WHO suggested larger trials with a greater variety of COVID-19 patients [83].

Favipiravir (FPV), a purine nucleic acid analog that targets RNA-dependent RNA polymerase (RdRP), is widely used as an oral anti-influenza drug [84]. Cai *et al*. conducted a clinical trial to evaluate the safety and efficacy of favipiravir in COVID-19 patients. A shorter viral clearance time as well as a significantly higher improvement rate in chest imaging was shown for the FPV group versus the LPV/r group. In addition, fewer adverse reactions were found in the FPV group than in the LPV/r group [85].

Arbidol, an important anti-viral drug candidate, also showed promising effects in COVID-19. Arbidol is a broad-spectrum antiviral molecule that inhibits both DNA as well as RNA viruses by altering the membrane structure of the virus [86]. According to Zhu *et al*., patients treated with arbidol show a shorter duration of positive RNA test compared to those treated with LPV/RTV, while nosignificant adverse effects are observed. The results indicated that arbidol monotherapy is superior to LPV/RTVin COVID-19 treatment [87]. Additionally, a retrospective cohort study showed that arbidol combined with LPV/RTV showed improved efficacy in COVID-19 patients [88]. Another retrospective, single-center study showed that the combination of Lianhuaqingwen and arbidol was effective for patients with mild symptoms within 5-7 days, and the cure rate was 98% [89]. Moreover, arbidol is considered to have a preventive effect. Yang *et al*. suggested that prophylactic oral arbidol was associated with a lower incidence of SARS-CoV-2 infection in medical staff [90]. Although accumulating evidence has demonstrated the potential clinical efficiency of arbidol, powered randomized control trials are still needed for further confirmation [91].

### Antisenescence drugs

Azithromycin and the closely related drug roxithromycin are macrolide antibiotics that can act as senolytic drugs that target and remove senescent cells [92]. Additionally, azithromycin is known to exert an antifibrotic effect by significantly extending the lifespan of myofibroblast cells. Moreover, azithromycin has been proven to reduce airway inflammation by inhibiting IL-6 and IL-1β expression in mouse models [93, 94]. Therefore, azithromycin is recommended by a number of researchers as a potential COVID-19 treatment strategy. However, Gbinigie *et al*. conducted a rapid review of the current literature. The results indicated that other than in the case of bacterial super infection, there was no evidence supporting the use of azithromycin for the treatment of SARS-CoV-2 infection outside of the context of clinical trials [95]. According to a clinical trial conducted by Rosenberg *et al*., receipt of azithromycin alone could not significantly reduce in-hospital mortality of COVID-19 patients [96].

Chloroquine (CQ) and its derivative hydroxychloroquine (HCQ) have the ability to induce alkalization, which functionally prevents the induction and accumulation of beta-galactosidase (β-Gal), known as a marker of senescence [97]. CQ and HCQ are widely used antimalarial and antiviral drugs and have recently received much attention as potential treatments for COVID-19 [98].CQ was recently demonstrated as an inhibitor ofSARS-CoV-2 in vitro, and the hydroxylated form, HCQ, has been proven to limit the replication of SARS-CoV-2 in vitro [99, 100]. Huang *et al*. found that CQ had a slight advantage over lopinavir/ritonavir in COVID-19 patients [101]. HCQ can also specifically inhibit the replication of SARS-CoV by interfering with the glycosylation of ACE2 [102]. Therefore, HCQ has been widely suggested as a potential treatment for patients with SARS-CoV-2 infection. A number of clinical trials have been conducted to explore the efficacies of CQ and HCQ. According to a small open-label nonrandomized clinical trial, HCQ treatment was significantly associated with viral load reduction in COVID-19 patients, and its efficacy could be reinforced by azithromycin [103]. However, according to a newly published comparative observational study among 181 COVID-19 patients with documented severe acute respiratory syndrome who required oxygen, HCQ did not show an obvious therapeutic effect. Additionally, eight patients in the HCQ group (10%) experienced electrocardiographic modifications that required discontinuation of treatment [104]. Despite the controversial results of these clinical trials, frequent side effects of CQ and HCQ, for example, worsening vision, nausea, digestive disorders and potential heart failure and even death, have hindered the wide application of these drugs [105]. Moreover, the results of a clinical trial conducted by Rosenberg *et al*., which involved 1438 COVID-19 patients, showed that compared with neither treatment, treatment with HCQ alone or combined with azithromycin was not significantly associated with differences in mortality [96].

Rapamycin is widely known as a key anti-aging drug and prevents the progression of senescence in human cell lines and animal models [106-108]. Rapamycin acts as an inhibitor of protein synthesis, inhibiting cytokine expression and viral replication [109]. In elderly patients, especially those with CHD or reduced T-cell counts, rapamycin can significantly reduce the expression of the serum senescence marker IL-6 [110]. As a candidate for potential use in COVID-19, rapamycin may prevent progression to severe forms of COVID-19 by downregulating the IL-6 pathway and reducing the number of senescent T-cells through the mTOR-NLRP3-IL-1β axis at the early stage of cytokine storms [111, 112]. Conversely, rapamycin is suggested as a novel intervention strategy beyond vaccines to prevent severe symptoms in COVID-19 [113]. Therefore, conducting clinical trials for rapamycin to prevent and treat the severity of COVID-19 patients, especially elderly patients, is strongly recommended.

### ACE2-related therapies

Some researchers have suggested that SARS-CoV-2 induces initial damage effects by downregulating ACE2 expression and blocking ACE2-mediated activity and activating ACE2 may be much more efficacious. Accumulating evidence has illustrated that the activation of ACE2 could be a positive treatment method. ACE2 activators are purported to have two therapeutic effects: avoiding the binding of the S protein of SARS-CoV-2 to ACE2 and promoting the protective effects of different organs, preventing lung injury and fibrosis [34, 114, 115].ACE2 activators, such as diminazene aceturate, are recommended for application in COVID-19 patients [34].

However, due to the positive-feedback loop between virus infection and ACE2 expression, some researchers have suggested the use of ACE2 inhibitors to block SARS-CoV-2 infection. Considering the role of ACE2 in maintaining organ functioning, especially in the lungs, the clinical application of ACE2 inhibitors in COVID-19 is under question [116]. The findings regardingACE2 have sparked a debate regarding the potential use of angiotensin-converting enzyme inhibitors (ACEIs) and AngII receptor blockers (ARBs) among elderly COVID-19 patients in the context of the pandemic [117]. However, after reviewing published relevant animal, in vitro and clinical studies, Chung *et al*. announced that the results did not show a higher risk of infection with ACEI or ARB use [118]. Considering the contradictory hypotheses and lack of scientific evidence and clinical data worldwide, the European Society of Cardiology and the American College of Cardiology recommended the continuation of ACEIs or ARBs for COVID-19 patients who were already taking these medicines [119, 120].

Human recombinant soluble ACE2 (hrsACE2) is an FDA-approved treatment, with a 2017 phase II trial for ARDS [121]. Circulating soluble ACE2 can bind to SARS-CoV-2 but is unable to inhibit cell infection [122]. According to Monteil *et al*., clinical grade hrsACE2 reduces SARS-CoV-2 recovery from Vero cells by 1,000-5,000 times, demonstrating that hrsACE2 can not only block SARS-CoV-2 infection but also protect against lung injury, suggesting a possible therapeutic approach [123].

### CD26 inhibitors

The clinical immune response to SARS-CoV-2 can be divided into 2 phases: an earlier phase of elimination by antiviral adaptive immunity and a later phase of damaged alveolar cells triggering innate inflammation [124]. According to Chen *et al*., the occurrence of ARDS may be associated with the later immune phase, and treatment to reduce inflammation during that phase may help reduce lung damage. CD26 inhibitors may potentially act to regulate an overactive immune reaction and prevent devastating lung injury [125]. In a mouse model of ARDS, which is the main cause of death in COVID-19 patients, CD26 inhibition by sitagliptin alleviated histological findings of lung injury by inhibiting the expression of IL-1β, TNF-α, and IL-6[126]. Birnbaum *et al*. reported that saxagliptin-mediated DPP4 inhibition could attenuate diabetes mellitus (DM) -induced activation of nod-like receptor family pyrin domain-containing 3 (NLRP3) inflammatory bodies, thus reducing serum CRP, TNF-α, IL-1β, IL-18 and IL-6 levels [127]. CD26 inhibitors, including sitagliptin, alogliptin, vildagliptin, saxagliptin, and linagliptin, are a class of drugs used effectively in the treatment of type 2 diabetes, which has demonstrated safety in elderly patients. These drugs have similar effects by binding to essentially the same catalytic site [128]. Research has shown that the administration of linagliptin can lead to a decrease in the concentration of proinflammatory factors, especially TNF-α and IL-6 [66]. Moreover, mathematical modeling has shown that the spread of MERS-CoV infection can be controlled by inhibiting the expression of CD26, with similar efficiency in SARS-CoV-2 infection [129]. Since the expression of CD26 is closely related to the pathogenesis and severity of COVID-19, CD26 inhibitors have recently been proposed as potential drugsagainst COVID-19 [130]. However, despite laboratory verification and theoretical feasibility, the actual efficacies of CD26 inhibitors in COVID-19 still require verification in further clinical trials.

### Immunosuppressive agents

As we mentioned above, COVID-19 severity and outcomes are closely related to the characteristics of the immuneresponse and subsequent cytokine storm incited by pathogenic T cells and inflammatory monocytes with a high level of IL-6 secretion, which are more severe in elderly patients [131]. Some researchers have recommended monoclonal antibodies that target IL-6,TNF-α and other cytokine pathways as a potential strategy to attenuate the inflammatory storm [132].

Tocilizumab is a marketed humanized monoclonal antibody that directly targets IL-6 receptors. Accumulating clinical trials have confirmed its safety and effectiveness in rheumatoid arthritis treatment. In a clinical trial among 45 patients, tocilizumab exerted a rapidly beneficial effect on fever and inflammatory markers in COVID-19 patients [133]. According to Xu *et al*., within 5 days after additional application of tocilizumab, the percentage of lymphocytes in peripheral blood returned to normal in 52.6% of severe COVID-19 patients, and all patients were discharged after anaverage of 15.1 days. The results indicated that tocilizumab could improve the clinical outcomes in severe COVID-19 patients by suppressing the immune response [131]. Recently, tocilizumab was approved for patients with severe SARS-CoV-2 pulmonary complications by the National Health Commission of the People’s Republic of China. Other monoclonal antibodies specifically targeting IL-6 pathways, such as sarilumab and siltuximab, are also recommended for COVID-19 treatment. Clinical trials are still needed to evaluate their efficacy and safety in COVID-19 patients.

The cyclosporine-cyclophilin A complex, including cyclophilin and tacrolimus, is considered to suppress organ rejection by halting the expression of TNF-α and IL-2 [134]. Moreover, according to Pfefferle *et al*., cyclophilin can block the replication of coronaviruses, including SARS-CoV, human CoV-229E and CoV-NL-63, feline CoV, among others. These results suggest that cyclophilin is a broad-spectrum coronavirus inhibitor and might be used for therapy in emerging coronavirus infections [135]. Therefore, the anti-inflammatory and antiviral properties of the cyclosporine-cyclophilin A complex make it a potential clinical application in severe COVID-19. However, considering the nephrotoxicity and hepatotoxicity of cyclophilin and tacrolimus, safety issues must be explored in COVID-19 patients, especially elderly individuals with liver and kidney involvement.

Corticosteroids show inhibitory effects on both innate and adaptive immune responses, as well as multiple types of immune cells [136]. Corticosteroids were among the first therapies tested in trials for preventing ARDS [137]. Meduri *et al*. constructed a clinical trial to investigate the efficacy of long-term corticosteroid treatment in patients with ARDS. The results showed that prolonged corticosteroid application could accelerate the resolution of ARDS and decrease hospital mortality [138]. Fadel *et al*. conducted a single pretest, single posttest quasi-experiment among 213 COVID-19 patients. The results indicated that an early short course of methylprednisolone application could reduce escalation of care and improve clinical outcomes in patients with moderate-to-severe COVID-19 [139]. However, clinical management of severe SARS and MERS patients revealed that corticosteroid therapy did not decrease mortality; in contrast, it caused delayed viral shedding [140]. In addition, Zha *et al*. announced that after analyzing the data of 31 COVID-19 patients, they found no association between corticosteroid therapy and outcomes in patients without ARDS [141]. The efficacy of corticosteroids in COVID-19 patients remains controversial. Thus, the Guideline for the Diagnosis and Treatment of COVID-19 (7^th^ version) recommended low-to-moderate doses and short courses of corticosteroid application in selected COVID-19 cases that represent excessive immune response activation or rapid progression of imaging changes

### MSC transplantation

MSCs are nonhematopoietic stem cells derived from the mesoderm, which can be isolated from various tissues, such as bone marrow, adipose tissue, umbilical cord blood, placenta, menstrual blood, dental pulp, and amniotic fluid [142]. Accumulating evidence from preclinical and clinical trials has demonstrated that MSCs have great advantages in anti-inflammation, anti-fibrosis and injury repair [143-145]. Shao *et al*. reported that MSCs could strongly suppress IL-6 production, thereby disrupting the development of cytokine storms [146]. Previously, it was demonstrated that MSC transplantation could significantly reduce the mortality of patients with H7N9-induced ARDS (17.6% died in the MSC group, whereas54.5% died in the control group). Moreover, no significant adverse effects were observed in these patients [147]. According to Leng *et al*., MSC transplantation could significantly improve pulmonary function and symptoms of COVID-19 patients without obvious adverse effects. Moreover, decreased TNF-α and increased IL-10 levels were detected in the MSC treatment group compared with the control group [148]. These results indicated that MSC-based therapy could be a potential alternative for managing patients with severe symptoms of COVID-19.

### Artificial liver system

The artificial liver system (ALS) is widely used as an effective support therapy in severe liver failure patients [149]. As mentioned above, the cytokine storm is the main motivator of COVID-19 progression and a poor prognosis, and its severity is closely associated with advanced age. Previously, clinical trials have demonstrated that the plasma exchange module of ALS exhibited high efficacy in attenuating the cytokine storm of H7N9 infection [150]. A single-center study showed that artificial liver plasma exchange could significantly reduce inflammatory cytokine levels, thus reducing mortality in critically ill patients with COVID-19 [151]. Our colleagues found that the application of ALS showed excellent prognosis in the treatment of severeCOVID-19 patients presenting a cytokine storm [152]. According to the Diagnosis and Treatment of COVID-19 (7th version) published in China, ALS is recommended in patients who exhibit rapid disease progression confirmed by lung imaging and a cytokine storm.

## Nutritional support in elderly people

To date, considerable evidence has demonstrated that food and nutrients could affect immune system function. Poor nutritional status is widely considered one of the significant risk factors for severe COVID-19 [153]. It has been directly highlighted that nutritional support may play an important role in determining COVID-19 outcomes [154]. Generally, there are nutritional deficiencies in calcium, vitamin C, vitamin D, folate, and zinc among the elderly population, especially among in-hospital patients [155]. According to a study in Wuhan, the prevalence of malnutrition is elevated in elderly patients with COVID-19 [156]. The researchers suggested that nutritional support should be strengthened during treatment. Malnutrition can exacerbate the immune system deficiency in elderly individuals, making them susceptible to SARS-CoV-2 infection [157]. A healthy, balanced diet can provide elderly individuals with necessary macro- and micronutrients, prebiotics, probiotics, and symbiotics to restore and maintain immune cell function, thus reducing the incidence of SARS-CoV-2 infection [153].

## Problems faced by the progress and application of the vaccine

The development of a safe and effective vaccine against SARS-CoV-2 for nonimmune individuals is an urgent and critical task for controlling the ongoing pandemic [68]. The developing coronavirus vaccines and drugs mainly target the spike glycoprotein or S protein, the major inducers of neutralizing antibodies. To date, Wei Chen *et al*. conducted a dose-escalation, single-center, open-label, nonrandomized, first-in-human trial of a recombinant adenovirus type5-vectored COVID-19 vaccine in Wuhan, China. The vaccine was tolerable and immunogenic 28 days after vaccination. The humoral responses against SARS-CoV-2 peaked at day 28 after vaccination in healthy adults, and rapid specific T-cell responses were noted from day 14 after vaccination [158].

However, even if a successful vaccine for SARS-CoV-2 becomes available, an individual’s immune response must be sufficiently strong to respond to the vaccine, and once exposure occurs, the reaction can later confer protection against the pathogen. Thus, vaccines cannot provide complete protection in elderly populations due to age-related declines in immune function and the accumulation of various diseases. According to Monto*et al*., influenza outbreaks still occur in elderly nursing homes even when vaccination rates reach 80-98% utilization [159]. Thus, the deficiency of the elderly group could become a gap in herd immunity that relies on vaccines. Some researchers recommended remodeling of the senescent immune system of elderly persons by allo-priming as a method to restore cellular immune function. Heterologous immunity can enhance the panviral protection upon each viral exposure, thereby providing long-term protection. The alloantigen priming strategy has been proposed in conjunction with viral-specific vaccines in the elderly population [160]. However, the efficiency requires further exploration.

Moreover, accumulating reports worldwide of some COVID-19 patients testing positive again after initially testing negative indicate the potential of re-infections and short-lasting immunity against COVID-19 [161]. To *et al*. firstly reported the case of COVID-19 re-infection by a phylogenetically distinct SARS-corona-virus-2 strain [162]. According to Ibarrondo *et al*., antibody loss in COVID-19 patients is quicker than that reported for SARS-CoV [163]. It means that humoral immunity against SARS-CoV-2 may not be long lasting in human bodies. Consequently, people doubt whether the vaccine could produce long-term immunity in the population.

## Conclusions

The COVID-19 pandemic suggests that we are facing a historic challenge to our capacity to protect the health of our elderly population. Protecting aging populations is now a central question in maintaining global health and biosecurity [4]. Lloyd-Sherlock *et al*. announced that due to great barriers in access to health services and support, older people in low-and middle-income countries are bearing the brunt of COVID-19 [164]. An open letter suggested that the WHO should act immediately to redress its neglect of older people, and member states must prioritize the needs of the elderly population in national responses and support for low- and middle-income countries [165]. The current results of biomedical, clinical and public health studies also highlight the need for therapy and prevention strategies for aging COVID-19 patients. We hope that this review can provide insight regarding the prevention and treatment of COVID-19 in elderly populations.
